# Adenosine 5′-monophosphate blocks acetaminophen toxicity by increasing ubiquitination-mediated ASK1 degradation

**DOI:** 10.18632/oncotarget.14059

**Published:** 2016-12-21

**Authors:** Xiao Yang, Yibei Zhan, Qi Sun, Xi Xu, Yi Kong, Jianfa Zhang

**Affiliations:** ^1^ Center for Molecular Metabolism, Nanjing University of Science and Technology, Nanjing, 210094, China; ^2^ School of Life Science and Technology, China Pharmaceutical University, Nanjing, 210009, China

**Keywords:** hepatotoxicity, APAP, 5′-AMP, JNK, ASK1

## Abstract

Acetaminophen (APAP) overdose is the most frequent cause of drug-induced liver failure in the world. Hepatic c-jun NH2-terminal protein kinase (JNK) activation is thought to be a consequence of oxidative stress produced during APAP metabolism. Activation of JNK signals causes hepatocellular damage with necrotic and apoptotic cell death. Here we found that APAP caused a feedback increase in plasma adenosine 5′-monophsphate (5′-AMP). We demonstrated that co-administration of APAP and 5′-AMP significantly ameliorated APAP-induced hepatotoxicity in mice, without influences on APAP metabolism and its analgesic function. The mechanism of protection by 5′-AMP was through inhibiting APAP-induced activation of JNK, and attenuating downstream c-jun and c-fos gene expression. This was triggered by attenuating apoptosis signal-regulated kinase 1(ASK1) methylation and increasing ubiquitination-mediated ASK1 protein degradation. Our findings indicate that replacing the current APAP with a safe and functional APAP/5′-AMP formulation could prevent APAP-induced hepatotoxicity.

## INTRODUCTION

Acetaminophen (APAP) is a widely used analgesic and antipyretic drug that is safe at therapeutic doses. However, when take at high doses or, rarely in particularly susceptible people at therapeutic doses, APAP can precipitate severe liver injury that can develop into acute liver failure [1–2]. In fact, APAP overdose is the most frequent cause of drug-induced liver failure in the some developed countries [3]. Although much APAP is metabolized via conjugation with glucuronic acid and sulfate and then excreted, a portion of APAP is converted by cytochrome P-450 metabolism to a reactive quinone form, N-acetyl-p-benzoquinone imine (NAPQI), which is inactivated by conjugation with glutathione (GSH) [4]. Once the pool of GSH is exhausted, any remained N-acetyl-p-benzoquinone imine covalently binds cellular macromolecules induces a series of molecular events that include alkylation of proteins, membrane lipid peroxidation, mitochondrial dysfunction, imbalance of intracellular calcium, formation of reactive oxygen species and reactive nitrogen species [5–6]. APAP-induced hepatocellular damage and necrotic and apoptotic cell death can result in severe centrilobular hepatotoxicity and acute liver failure [7–8].

In APAP-induced liver injury, hepatic JNK activation is thought to be a consequence of oxidative stress produced during APAP metabolism [9]. Inhibiting or silencing expression of JNK1 and JNK2 markedly protected the liver against APAP-induced injury, despite extensive GSH depletion and covalent binding caused by the production of NAPQI [10–11]. Recently, JNK was reported to play a critical role in APAP-induced hepatotoxicity in mice [12–13]; thereby identifying that inhibition of JNK can be used as important therapeutic way in the treatment of APAP-induced acute liver failure. JNK is activated by sequential protein phosphorylation through a mitogen-activated protein kinase (MAPK) module. Several MAPK kinase kinases (MAPKKKs) have been identified in the JNK cascade, such as apoptosis signal-regulated kinase 1 (ASK1) [9, 14].

Adenosine 5′-monophosphate (5′-AMP) is a natural molecule of adenosine triphosphate metabolism. Administration of exogenous 5′-AMP displays multiple regulatory functions and important physiological roles [15–17]. In the present study, we identified that co-administration of APAP and 5′-AMP ameliorated significantly APAP- induced hepatotoxicity. The mechanism of protection by 5′-AMP was through inhibiting JNK signaling pathway and protein modification. Our results suggest that replacing the current APAP with a safe and functional APAP/5′-AMP formulation could prevent APAP-induced hepatotoxicity.

## RESULTS

### Co-administration of APAP and 5′-AMP ameliorated APAP-induced hepatotoxicity

**Figure 1:**
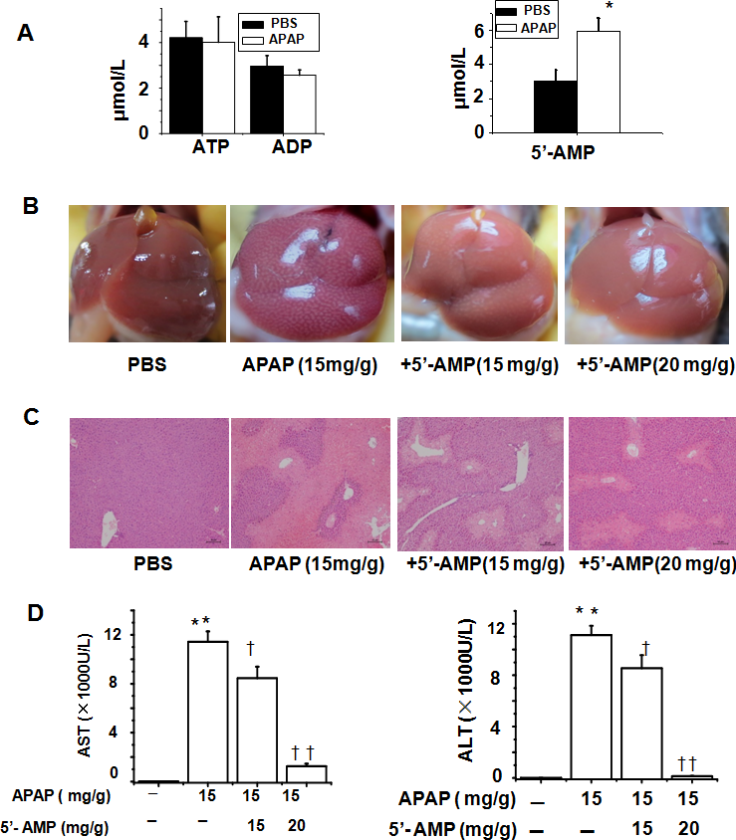
Co-administration of APAP and 5′-AMP attenuates APAP-induced hepatotocixity (**A**) Plasma nucleotides were determined by HPLC at 24 h after PBS or APAP (15 mg/g ) administered intragastrically. (**B**) Macroscopic appearance of representative liver samples at 24 h after treatment. (**C**) Representative H&E-stained liver sections of mice at 24 h after APAP (15 mg/g) or co-administration of APAP (15 mg/g) and 5′-AMP (15 mg/g and 20 mg/g, respectively). Bar = 10 μm. (**D**) Serum activities of AST and ALT at 24 h after APAP administered intragastrically. Data are expressed as mean ± SEM. *P < 0.05, **P < 0.01, compared with PBS group; ^†^*P* < 0.05, ^††^*P* < 0.01 compared with APAP group (*n* = 5).

To identify possible changes in extracellular nucleotides and their possible role in APAP-induced hepatotoxicity, we looked for differences in circulatory nucleotides between APAP-treated and control mice. HPLC analysis from these samples indicated that the plasma 5′-AMP, but not ATP and ADP, was elevated in APAP-treated mice compared to control mice (Figure 1A). We hypothesized that increased 5′-AMP was a feedback protection against APAP-induced liver injury. Then, wild-type mice were intragastrically challenged with APAP (15 mg/g) or co-administration of APAP and 5′-AMP. Interestingly, the protective effects of 5′-AMP (15, 20 mg/g) was detectable macroscopically on liver appearance, with strong hepatic injury changes in livers derived from APAP-treated mice, but normal liver morphology in the mice treated with co-administration of APAP and 5′-AMP (Figure 1B). Histological analysis by H&E staining showed hepatocyte necrosis in the livers of APAP mice, whereas co-administration of APAP and 5′-AMP attenuated the area and extent of necrosis (Figure 1C). Consistent with their reduced histological liver damage, mice treated with APAP showed evidence of severe liver injury at 24 h as indicated by the significant increase of serum AST and ALT levels, and co-administration of 5′-AMP and APAP resulted in a significant reduction in serum AST and ALT values compared with single APAP groups (Figure 1D). Next, we found that the protective effects of 5′-AMP was completely lost while mice were given 5′-AMP at 1 h after APAP (data not shown). Together, these results revealed that co-administration of 5′-AMP and APAP significantly ameliorated APAP-induced hepatotoxicity.

### 5′-AMP failed to change APAP metabolism and analgesic function

**Figure 2:**
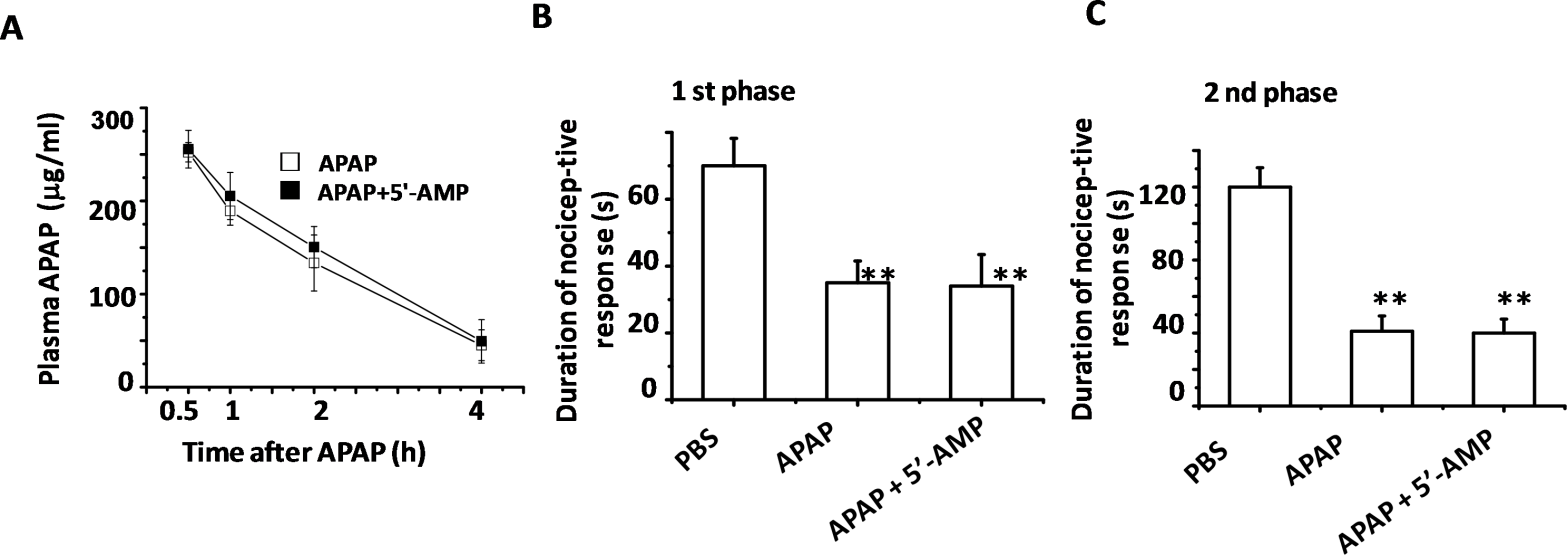
5′-AMP failed to influence APAP catabolism and analgesic Mice were administered intragastrically with APAP or Compound APAP. Livers were collected at indicated time after APAP (15 mg/g) or compound APAP (plus 5′-AMP, 20 mg/g) treatment. (**A**) Plasma APAP concentration was determined by HPLC (*n* = 15). (**B**) The cumulative response time of licking and biting the injected paw was measured during the period of 0–5 min (1st phase), and (**C**) 20–40 min 2nd phase. Mice were treated orally once with APAP or the compound for 30 min prior to the formalin (1%, 10 μl) injection into left hind paw subcutaneously. Data are expressed as mean ± S.E.M. *P < 0.05, **P < 0.01, compared with PBS group (*n* = 5).

To investigate whether 5′-AMP influences APAP metabolism, we preformed a HPLC analysis for plasma APAP level with time course after APAP injection. 5′-AMP failed to change APAP degradation rate during 0.5–4 h after APAP administration. Statistics analysis revealed that there are no significant differences of APAP degradation rate between two groups (Figure 2A). APAP is a widely used over-the-counter analgesic drug. We used formalin- induced nociceptive behavioral test to investigate whether 5′-AMP attenuated the analgesic effect of APAP. Either pretreatment with APAP or APAP plus 5′-AMP markedly reduced the cumulative response time of formalin responses in both 1st phase and 2nd phase. (Figure 2B, 2C), indicating 5′-AMP had no effect on analgesic function of APAP.

### 5′-AMP protected against APAP-induced hepatocellular damage *in vitro*

**Figure 3:**
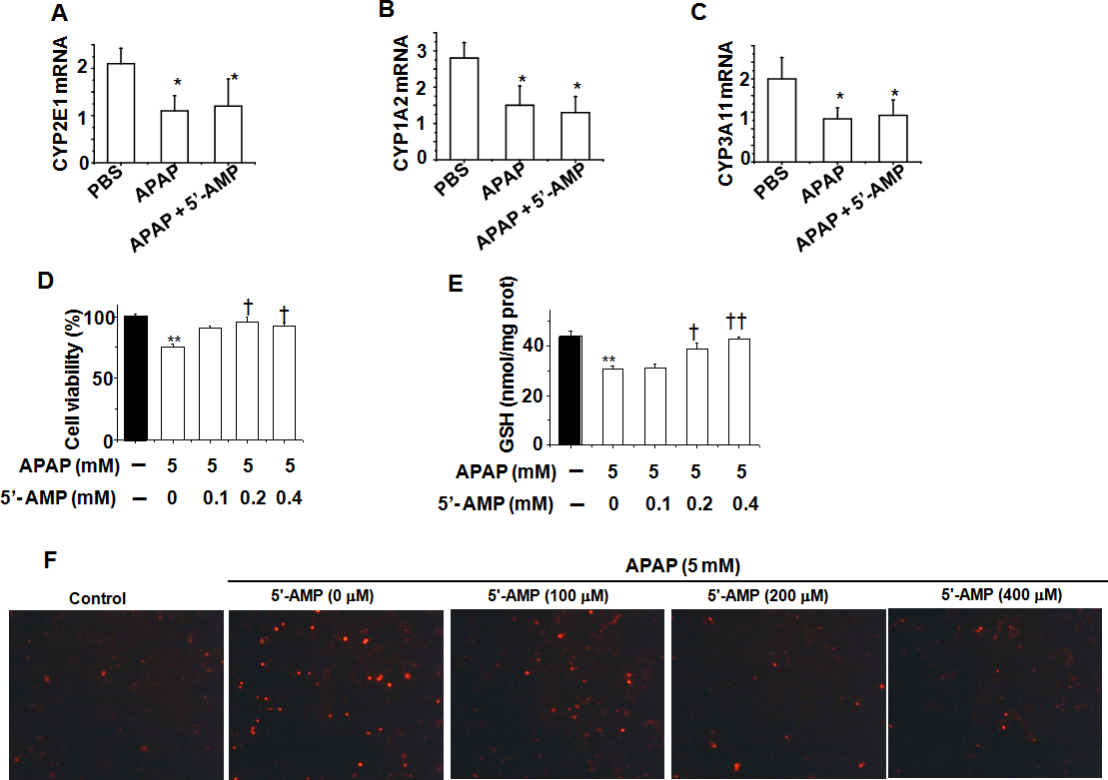
5′-AMP protected against APAP-induced cellular damage in liver cell L02 The hepatic mRNA expressions of *CYP2E1* (**A**), *CYP1A2* (**B**), and *CYP3A11* (**C**) were measured by quantitative RT- PCR at 6 h after APAP treatment. Mice were administered intragastrically with APAP (15 mg/g) or compound APAP (plus 5′-AMP, 20 mg/g), and liver samples were collected at 6 h after treatment (*n* = 5). (**D**) Liver cell L02 was treated with APAP (5 mM) in the presence or absence of 5′-AMP (0.1mM, 0.2 mM, 0.4 mM). (D) Cell viability was determined by MTT assay after 24 h. (**E**) Intracellular GSH was measured by GSH assay kit after 24 h. Data are expressed as mean ± S.E.M. of three independent experiments. *P < 0.05,**P < 0.01, compared with PBS group; ^†^*P* < 0.05, compared with APAP group. (**F**) Representative images of phase contrast with PI staining of L02 cells treated APAP for 24 h. Original magnification: ×100.

Cytochrome P-450 enzymes plays a major role in the conversation of APAP into hepatotoxic N-acetyl-p-benzoquinoneimine (NAPQI) [18]. Treatment with 5′-AMP had no influence on the expression of P450 enzymes mRNA (Figure 3A–3C). Next, we used human hepatic L02 cells to assess the effect of 5′-AMP on APAP-induced hepatocellular damage. Cell viability was recovered in 5′-AMP-treated cells after APAP exposure (Figure 3D) and the APAP-induced depletion of GSH in 5′-AMP- treated cells were significantly diminished (Figure 3E). Furthermore, compared with control group, APAP induced hepatocellular cell death in L02 cells, and 5′-AMP markedly suppressed cell death (Figure 3F) with a dose-dependent manner.

### 5′-AMP inhibited APAP-induced JNK activation

**Figure 4:**
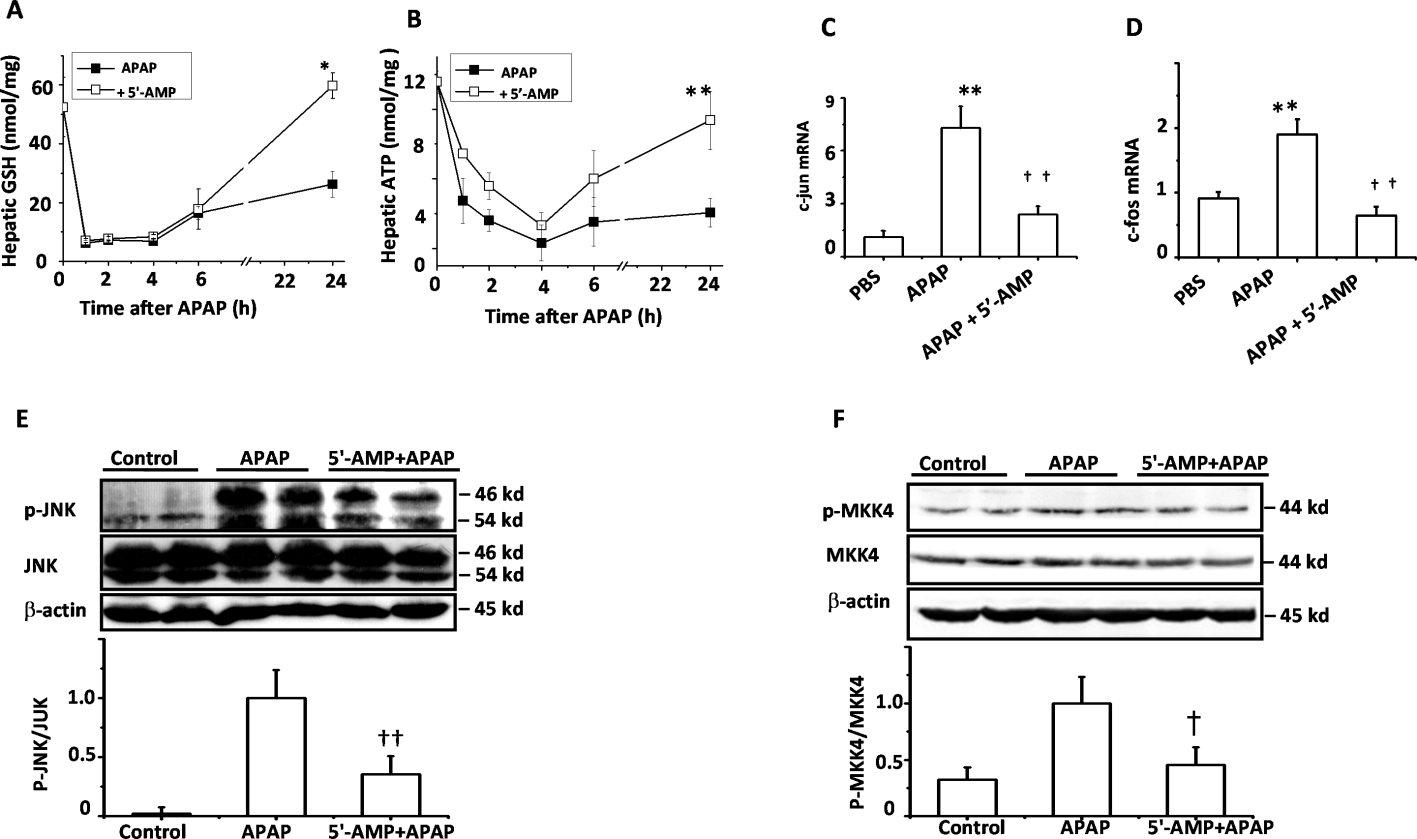
5′-AMP protected from GSH and ATP depletion and suppressed JNK activation Mice were administered intragastrically with APAP (15 mg/g) or co-administration of APAP (15 mg/g) and 5′-AMP (20 mg/g). Livers were collected at indicated time after treatment. (**A**) Total liver homogenate GSH were measured by GSH assay kit and (**B**) Liver ATP were assayed by HPLC (*n* = 5). (**C**, **D**) The hepatic mRNA expressions of c-jun and c-foswere measured by quantitative RT- PCR at 6 h after APAP treatment (*n* = 5). (**E**, **F**) Activated JNK and MKK4 were measured by western blotting 3 h after treatment. β-actin was used as a loading control, The bands were quantified by a Gel analysis software (*n* = 3). Data are expressed as mean ± SEM. *P < 0.05, **P < 0.01, compared with PBS group; ^†^*P* < 0.05, ^††^*P* < 0.01 compared with APAP group.

The depletion of GSH by NAPQI is an important component of APAP-induced liver injury [19]. The results of GSH measurement demonstrated that the protective effects of 5′-AMP against APAP-induced liver injury was not due to inhibiting GSH consumption in early stage. At 1 h after APAP, the bulk of hepatic GSH was depleted to 85%, displaying equivalent depleted rate in both groups. However, the hepatic GSH was significantly higher in 5′-AMP-treated mice at 24 h after APAP treatment (Figure 4A). It is well established that APAP hepatotoxicity causes mitochondrial dysfunction with depletion of hepatic ATP levels [20–21]. Using HPLC, we compared changes in ATP levels with alterations in energy metabolism. We chose to evaluate these parameters at different time points during the initiation of the injury and late time when substantial was evident. ATP levels in the livers of both mice treated with APAP and APAP plus 5′-AMP were robustly reduced at the early stage. Treatment with 5′-AMP did not result in an early ATP recovery but improved the energy status at the late time point (24 h) (Figure 4B). JNK activation is an early key signal in mediating mitochondria-mediated lethal cell triggered by toxicants in hepatocytes [22–23]. Therefore, we investigated whether APAP-induced JNK activation was attenuated by 5′-AMP. First, because c-jun and c-fos genes are known to be JNK-dependent genes and reported to be associated with the degree of APAP-induced- liver injury, we examined the c-jun and c-fos mRNA expression in liver at 6 h after APAP administration. Expressions of c-jun and c-fos mRNA were significantly elevated in APAP mice and suppressed by 5′-AMP treatment (Figure 4C, 4D). Furthermore, we used western blotting to examine the time course of JNK activation (phosphorylation), demonstrated JNK activation reached a peak plateau at around 3 h after APAP treatment (data not shown). Then we examined APAP-induced JNK activation in 5′-AMP treated mice liver 3 h after APAP administration. Treatment of mice with 5′-AMP significantly decreased the levels of phospho-JNK while the total JNK levels were unaffected (Figure 4E). Similar to JNK, MKK4 activation was attenuated by 5′-AMP treatment (Figure 4F).

### 5′-AMP attenuated ASK1 level through protein modification

**Figure 5:**
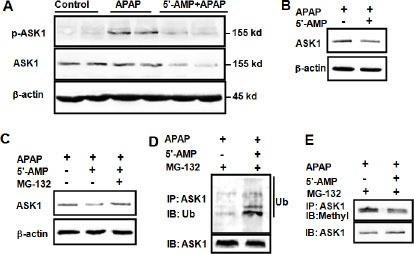
5′-AMP influenced ASK1 modification and degradation Mice were administered intragastrically with APAP (15 mg/g) or co-administration of APAP (15 mg/g) and 5′-AMP (20 mg/g). (**A**) Activated ASK1 and total ASK1 expression were measured by western blotting 3 h after treatment. β-actin was used as a loading control. (**B**) L02 cells were treated with APAP (5 mM) in presence and absence of 0.4 mM 5′-AMP for 2 h, (**C**) L02 cells were treated with APAP (5 mM) in presence and absence of 0.4 mM 5′-AMP, 10 μM MG132 for 2 h. ASK1 was determined by western blotting and β-actin was used as a loading control. ASK1 was immunoprecipitated, the immunocomplexes were resolved by SDS-PAGE, and ubiquitinated ASK1 (**D**) and methylated ASK1 (**E**) were detected by western blotting with anti-ubiquitin and anti-mono and dimethyl Arginine. Three independent experiments were performed and representative blotting were shown.

MKK4 activation is regulated by phosphorylation of ASK1. We examined whether APAP induced ASK1 activation in control mice, APAP-treated mice, and APAP plus 5′-AMP-treated mice. ASK1 activation was observed at 3 h after APAP administration and was significantly attenuated by 5′-AMP. Unexpectedly, 5′-AMP significantly decreased the total ASK1 expression (Figure 5A) in livers. In L02 cells, total ASK1 expression was also significantly attenuated by 5′-AMP (Figure 5B). Furthermore, the loss of ASK1 in 5′-AMP-treated cells was prevented by MG-132 (a Proteasome inhibitor), indicating that it was undergoing proteasome-mediated degradation (Figure 5C). To determine whether 5′-AMP affects ASK1 protein stability, we examined the ubiquitination and methylation of ASK1. The high molecular bands are usually ubiquitination of target proteins for degradation [24]. Indeed, these high molecular mass above ASK1 protein were shown to be polyubiquitinated (Ub) ASK1 proteins as demonstrated by immunoprecipitation with anti-ASK1 followed by western blot with anti-Ub. The basal ubiquitination of ASK1 was detected and 5′-AMP treatment significantly increased ASK1 ubiquitination (Figure 5D). Following investigation revealed that ASK1 methylation was significantly attenuated by 5′-AMP (Figure 5E).

## DISCUSSION

Previous findings demonstrate that 5′-AMP inhibit inflammatory response and ameliorate LPS-induced liver injury [17]. In the present study we found APAP treatment caused a feedback increase in plasma 5′-AMP level, and revealed a novel protective role for 5′-AMP against APAP-induced hepatotoxicity. APAP treatment resulted in profound liver cell injury and correlated with the rise in serum AST and ALT levels. Cell injury inevitably caused intracellular nucleotides to be released into the circulation [25]. Blood cell-based CD39 mediates conversion of ATP and ADP to 5′-AMP, whereas soluble 5′-nucleotidase and alkaline phosphatase catabolize 5′-AMP to adenosine. Elevated plasma 5′-AMP after APAP treatment implied that 5′-AMP involved in APAP-induced liver injury. Indeed, 5′-AMP treatment decreased the phosphorylation of JNK, MKK4, and ASK1. JNK activation is a major component of liver injury and is activated during hepatocyte injury caused by, APAP, bile acids, concanavalin A, and ischemi-reperfusion injury [26–27]. C-jun and c-fos have been reported to be associated with the degree of APAP-induced liver injury [28]. Because these genes encode components of the JNK activating pathway has been reported to mediate hepatocyte death, activation of the JNK pathway is considered to promote APAP-induced liver injury [29]. ASK1 is known to associate with redox-sensitive proteins such as thioredoxin-1, which inhibits kinase activation [30]. ASK1 deficiency protected mice from APAP-induced liver injury and reduced JNK activation, indicating that ASK1 acts as upstream of MAP3K in this process [9].

5′-AMP also decreased the total ASK1 expression, which was related to 5′-AMP- induced hyper-adenosine. Adenosine inhibited AdoHcy hydrolysis *in vitro* and *in vivo*. Inhibition of AdoHcyase results in the intracellular accumulation of AdoHcy and thus potent inhibition of some AdoMet- dependent methyltransferases [31]. The ratio of AdoMet to AdoHcy levels is used frequently as an indicator of cellular methylation capacity, whereby a decrease in this ratio predicts reduced cellular methylation potential [32]. Methylated proteins were seen to have significantly longer half-life than proteins for which no methylation was found [33]. The functions of methylated proteins are co-regulated by ubiquitination, phosphorylation or other post- translational modifications [33]. In our observation, 5′-AMP attenuated the methylation of ASK1 and enhanced the ubiquitination of ASK1, which accelerate ASK1 degradation, leading to an inhibition of MAPKs cascade. Previous research indicated that ASK1 methylation deceased the H_2_O_2_-induced activity of ASK, impairing stress-induced signaling that controls a variety of cellular events including apoptosis. [34–35].

In summary, the present study is the first to demonstrate a significant protective effect of 5′-AMP against APAP-induce hepatotoxicity in mice. Findings from the current study suggest that 5′-AMP plus APAP represent a promising formulation in lowering APAP-induced hepatotoxicity.

## MATERIALS AND METHODS

### Animals

Male wild-type C57BL/6J mice were used at 8–10 weeks of age with a body weight of 20–25 g in this work. Mice were maintained under standard laboratory conditions, with full access to food and water *ad libitum*, and 12 h light/ 12 h dark (LD) cycles with lights on at 7:00 a.m. and off at 7:00 p.m. All experiments were in accordance with the guidelines of the Animal Care and Use Committee at Nanjing University of Science & Technology.

### Drug administration and experimental design

Acetaminophen (Sigma-Aldrich) was dissolved in warm phosphate-buffered saline (PBS, 50°C) and cooled to 37°C before injection of mice. Mice were fasted overnight (16–18 hours) before administration of a single dose of acetaminophen (300 mg/kg, body weight) together with or without 5′-AMP. All mice were sacrificed at the indicated time periods. The blood was collected from the carotid artery and the liver of each mouse was removed immediately and then was kept at −80°C until analyzed.

### Serum biochemistry assay and histological analysis

Activities of serum aspartate transaminase (AST) and alanine transaminase (ALT) were measured using an AU2700 automatic biochemical analyzer (Olympus, Tokyo, Japan). For histopathological analysis, liver tissue was fixed in 10% phosphate buffered formalin and paraffin embedded, and cut into 4 μm sections. Sections were stained with hematoxylin and eosin (H&E) and analyzed by light microscopy.

### Cell culture and cell treatment

Human liver cell line L02 were maintained in supplemented DMEM in an atmosphere at 90% humidity containing 5% CO_2_ at 37°C. At the end of the preincubated period, cells were rinsed with PBS, and the medium was exchanged to DMEN without fetal bovine serum. The cells were treated with APAP (5 mM) in the presence or absence of 5′-AMP (100 μM, 200 μM, 400 μM). CGS15943 (100 nM) was stimulated 30 min before APAP. Cell viability was determined by the MTT assay. Cell death was determined by exposing the L02 cells to a solution containing the DNA-binding dye propidium iodide (PI). PI enters those cells with a damage membrane, staining the DNA red. Cells were then observed under a fluorescence microscope.

### RNA isolation and quantitative RT-PCR

Total RNA was extracted from fresh liver samples with Trizol (Invitrogen, Carlsbad, CA) according to the manufacturer's instructions. 20 ng RNA was converted to cDNA using reverse transcript enzyme (Invitrogen, Carlsbad, CA). Quantitative RT-PCR was performed, and results were analyzed using an ABI 7300 Detection System utilizing SYBR Green dye (Toyobo, Osaka, Japan). All primer sequences used for quantitative RT-PCRs are shown in Table 1. Relative gene expression in comparison with Gapdh expression was calculated by the comparative cycle threshold (CT) method.

**Table 1:** Primer sequences for quantitative RT-PCR analysis

Gene	Forward/Reverse	Primer (5′ to 3′)
*c-Jun*	Forward	5′-AATCAGACAGGGGACACAGC-3′
	Reverse	5′-GAAAAGTAGCCCCCAACCTC-3′
*c-Fos*	Forward	5′-TGGCACTAGAGACGGACAGA-3′
	Reverse	5′-TCCTACTACCATTCCCCAGC-3′
*ASK1*	Forward	5′-TCGCACTCCAAGATGGTAAA-3′
	Reverse	5′-CATTTCGGGAAGCTGGACT-3′
*CYP2E1*	Forward	5′-CTTAGGGAAAACCTCCGCAC-3′
	Reverse	5′-GGGACATTCCTGTGTTCCAG-3′
*CYP1A2*	Forward	5′-AAAGGGGTCTTTCCACTGCT-3′
	Reverse	5′-AGGGACACCTCACTGAATGG-3′
*CYP3A11*	Forward	5′-GGGGGACAGCAAAGCTCTAT-3′
	Reverse	5′-TTCTGTCTTCACAAACCGGC-3′
*GAPDH*	Forward	5′-CATCCACTGGTGCTGCCAAGGCTGT-3′
	Reverse	5′-ACAACCTGGTCCTCAGTGTAGCCCA-3′

### Immunoprecipitation

L02 cells after various treatments were washed twice with cold PBS and lysed in cold lysis buffer containing 50 mM Tris-HCl, pH 8.0, 150 mM NaCl, 1% NP-40, 0.5% sodium deoxycholate, 0.1% SDS, 2 mM PMSF and fresh protease inhibitors. The lysates were precleared with protein A-Sepharose beads for 1 h at 4°C. Then lysates were incubated with the immunoprecipitating antibody for 1 h on ice, protein G- Sepharose beads were added on rotator at 4°C overnight. The beads were washed four times with lysis buffer and heated at 95°C for 5 min in sample buffer.

### Immunoblotting

Preparation of total protein extracts from mice liver or cells was performed following the procedure described previously [36]. The extracted proteins and immunoprecipitates were separated by SDS-PAGE 10% or 12% polyacrylamide gel and then electrically transferred to a PVDF membrane. After blocking with 5% (w/v) BSA in TBST at room temperature for 1 h, the membranes were then incubated with an appropriate specific primary antibody (Anti-JNK1/2, anti-phospho JNK1/2, anti- MKK4, anti-phospho MKK4, anti-ASK1, anti-phospho ASK1, anti-Ubiquitinin, Cell Signaling Technology, Boston; Anti-mono and dimethyl Arginine, Abcam, Cambridge, MA, USA) at 4°C overnight, followed by incubation with HRP-conjugated secondary antibody (1:15,000; Sunshine Biotechnology) and detected by enhanced chemical luminescence kit (Thermo scientific, Hudson, NH, USA). The quantification of the bands was performed by the Gel Analysis V2.02 Software (Clin Science Instruments, Shanghai, China).

### Intracellular GSH

Preparation of cell or liver lysates was performed as described previously [6]. Reduced intracellular GSH levels were measured using a GSH assay kit (Jianchen, Nanjing, China) according to the manufacturer's instructions.

### HPLC analysis

Plasma APAP concentration was determined by HPLC as described previously [37–38], S-adenosylmethionine (AdoMet), S-adenosylhomocysteine (AdoHcy), 5′-AMP, ADP and ATP were extracted from liver samples and cells using 0.4 N perchloric acid and analyzed by HPLC, as described previously [17]. Extracts were seperated and quantified using reverse- phase HPLC (Waters 1525 system; Millipore, Bedford,MA) on a Partisphere bounded phase C18 (reverse phase) cartridge column. Pure APAP, 5′-AMP, ADP and ATP (Sigma-Aldrich) were used to identify the peaks and obtain the calibration curves.

### Formalin treatment and nociceptive behavioral analysis

Formalin-induced nociceptive test was performed as described previously [39]. 10 μl of 1% formalin solution, made up in physiologic normal saline, was injected subcutaneously (s.c.) under the surface of the left hindpaw. Mice were treated orally once with APAP (15 mg/g) together with or without 5′-AMP (20 mg/g) for 30 min prior to the formalin injection. Mice were observed simultaneously from 0 to 40 min following formalin injection. The early phase of the nociceptive response normally peaked 0 to 5 min after formalin injection and the late phase 20 to 40 min after formalin injection, representing the direct effect on nociceptors and inflammatory nociceptive responses, respectively. The time spent licking and biting the injected paw was measured.

### Statistics

The data were expressed as the mean ± S.E. Comparison between two groups was performed with the student *t* test or one-way ANOVA, followed by Tukey's *post hoc* test for multiple groups. *P* < 0.05 was considered significant.

## Abbreviations

5′-AMP: adenosine 5′-monophosphate
APAP: acetaminophen
i.p.: intraperitoneal
i.g.: intragastrical
AST: aspartate transaminase
ALT: alanine transaminase
Ado: adenosine
S-adenosylmethionine: AdoMet
S-adenosylhomocysteine: AdoHcy
JNK: c-jun NH2-terminal protein kinase
ASK1: apoptosis signal-regulated kinase 1
MAPK: mitogen-activated protein kinase
MKK4: MAP kinase kinase
NAPQI: N-acetyl-p-benzoquinone imine
